# Using lake sediments to assess the long-term impacts of anthropogenic activity in tropical river deltas

**DOI:** 10.1177/20530196231204334

**Published:** 2023-10-09

**Authors:** Richard E Walton, Heather L Moorhouse, Lucy R Roberts, Jorge Salgado, Cai JT Ladd, Nga Thu Do, Virginia N Panizzo, Pham Dang Tri Van, Nigel K Downes, Duc Anh Trinh, Suzanne McGowan, Sarah Taylor, Andrew CG Henderson

**Affiliations:** 1 Newcastle University, UK; 2 Earthwatch Europe, UK; 3 University of Nottingham, UK; 4 Aarhus University, Denmark; 5 UCL, UK; 6 University of Glasgow, UK; 7 Electric Power University, Vietnam; 8 Can Tho University, Vietnam; 9 Vietnam Atomic Energy Institute, Vietnam; 10 Netherlands Institute of Ecology The Netherlands

**Keywords:** multiple stressors, palaeolimnology, resource management, river deltas, tropics

## Abstract

Tropical river deltas, and the social-ecological systems they sustain, are changing rapidly due to anthropogenic activity and climatic change. Baseline data to inform sustainable management options for resilient deltas is urgently needed and palaeolimnology (reconstructing past conditions from lake or wetland deposits) can provide crucial long-term perspectives needed to identify drivers and rates of change. We review how palaeolimnology can be a valuable tool for resource managers using three current issues facing tropical delta regions: hydrology and sediment supply, salinisation and nutrient pollution. The unique ability of palaeolimnological methods to untangle multiple stressors is also discussed. We demonstrate how palaeolimnology has been used to understand each of these issues, in other aquatic environments, to be incorporated into policy. Palaeolimnology is a key tool to understanding how anthropogenic influences interact with other environmental stressors, providing policymakers and resource managers with a ‘big picture’ view and possible holistic solutions that can be implemented.

## Introduction

Tropical river deltas are dynamic and productive ecosystems that sustain livelihoods and have been important in the development of civilisation. Deltas form through the deposition of sediment and nutrient inputs from both the upstream catchment by freshwater flow and from the nearshore environment by the tide, creating fan-like landscapes intersected by a distributary channel network (Hiatt and Passalacqua, 2015; Tockner et al., 1999). Deltas are often referred to as socio-ecological systems (SESs) (Berkes and Folke, 1998; Colding and Barthel, 2019; Kuenzer and Renaud, 2012) because of the interconnected relationships between human populations and the ecosystem services provided by numerous habitats from upstream catchments to the coast. Even though tropical deltas are important centres of natural resource extraction, agriculture, aquaculture and industry (Adams et al., 2018; Adekola and Mitchell, 2011; Kuenzer and Renaud, 2012), they are facing mounting pressures from anthropogenic activities and environmental change that jeopardise human populations and the vital ecosystem services they provide (Islam et al., 2015; Kuenzer and Renaud, 2012).

Anthropogenic influence in any part of the watershed can lead to issues downstream and throughout the delta. For example, the construction of dams along the Mekong River basin has reduced freshwater flow to downstream Vietnam, resulting in increased saline intrusion within coastal waterways and soils (Li et al., 2017; Eslami et al., 2019). Sand mining, in addition to dam construction, reduces sediment flux downstream and increases riverbank and coastal erosion (Hackney et al., 2020; Jia et al., 2007; Marchesiello et al., 2019). Widespread nutrient enrichment arising from agricultural intensification (e.g. fertiliser runoff) and heavy metal pollution by industrial or urban discharges into surrounding waterways has led to water quality degradation (Downing et al., 1999; Ezekwe and Edoghotu, 2015; Rahman et al., 2011). Environmental degradation due to anthropogenic influences negatively **impact** fisheries, drinking water and biodiversity (Baran et al., 2011; Benneyworth et al., 2016; Winemiller et al., 2016) and increases susceptibility to climate change effects (Ngo et al., 2018; Thilakarathne and Sridhar, 2017).

To address these interacting challenges, governments often try to balance economic development and environmental protection by instituting management policies for the sustainable use of delta resources. However, a lack of long-term monitoring (over decades to centuries – most regular monitoring does not precede the 1990s) (Davidson et al., 2013; Smol, 1992) means it is difficult to detect changes in delta biogeochemistry, ecological regimes, or driving stressors, hampering appropriate policy responses. In the absence of lengthy water quality, sediment transport and hydrological data, palaeolimnology – the study of past lake and wetland sediments to reconstruct historical limnological and climatic conditions – can provide long-term perspectives of natural baselines (i.e. prior to a disturbance) and variability within ecosystems, as well as the impacts humans are having on the environment including the effectiveness of existing policy or management regimes.

**Figure 1.**
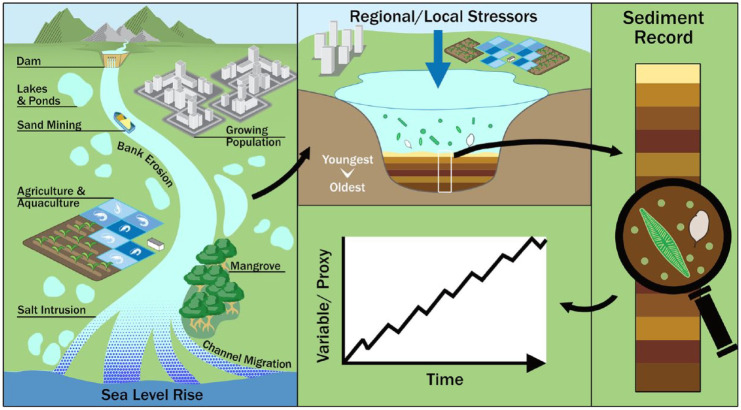
Stressors on river delta systems may be better understood using palaeolimnology. This diagram illustrates various stressors that may have significant impacts on delta systems, but sediments accumulating in aquatic sediments may hold clues as to how the system is responding over time, and if the systems are responding to management plans aimed at mitigating anthropogenic impacts.

Palaeolimnology uses chronologically controlled (e.g. radioisotope dating – ^210^Pb, ^137^Ams, ^14^C) sediment cores to provide temporal context in the study of organic and inorganic remnants found in sediments (referred to as proxies) to uncover the historical environmental conditions of aquatic systems and their catchments over timescales of decades to millennia (Figure 1). Palaeolimnological proxies have been used successfully to detect shifts in the biological, environmental and physical conditions within some tropical aquatic ecosystems (Velez et al., 2018; Wengrat et al., 2018; Zong et al., 2010a). Such shifts can be attributed to specific events or activities (e.g. dam construction, climate change, land-use change (Bianchi et al., 2013; Inda et al., 2016; Zeng et al., 2018)) and provide a valuable resource for setting restoration goals and management strategies (Alexson et al., 2018; Köster et al., 2007; Perga et al., 2015). The power of proxies to detect long-term environmental and ecological change, and to elucidate the complex dynamics existing between humans and deltaic environments, have led to palaeolimnological methods being used to inform tropical delta management plans (Escobar et al., 2020). Palaeolimnology can also demonstrate the timescales over which changes within a system occur; from long-term shifts in hydrology to short-term pollution impacts, as well as understanding contemporary or predicted conditions (e.g. land-use change, climate change) in various aquatic systems within the context of historical baselines (Bennion et al., 2011). Here, we argue that policymakers and resource managers in tropical nations should engage with the use of palaeolimnology more frequently to better understand the changes mega-deltas are undergoing and work for greater sustainability within these systems.

In this review, we aim to highlight how palaeolimnology can be used to inform management options for three key issues faced by tropical delta systems: (1) hydrology and sediment supply, (2) salinisation of freshwaters and soils and (3) nutrient enrichment and pollution of waterways. We discuss how palaeolimnology has already contributed to our understanding of these issues across different geographical regions, and how it has contributed to resource management in other environmental settings, and explaining how its inclusion can serve to strengthen future management plans in tropical delta systems. We argue for palaeolimnology to be integrated as a key management tool for governments, policymakers and resource managers, alongside robust routine monitoring.

## Hydrology and sediment supply

Central to the functioning of delta environments is hydrology (Dada et al., 2018; Le et al., 2007) and sediment flux (Coleman and Wright, 1975; Rahman et al., 2018), which sustain aquatic ecosystems and processes of land accretion or erosion. Human modifications such as dam construction (Mikhailov et al., 2015; Xue et al., 2011), channel alterations (Restrepo and Kettner, 2012; Zhang et al., 2009) and land-use change (Remondi et al., 2016; Setti et al., 2020) can drastically transform flooding frequency and occurrence. These modifications can reduce freshwater supply to deltas, allow tidal flow of seawater further inland (Eslami et al., 2019; Gole and Vaidyaraman, 1967) and affect sediment flow, to cause excessive accretion or erosion in different regions of a delta. Excessive riverbed siltation can increase water levels that risk overtopping artificial embankments surrounding low-lying, reclaimed land to keep out river water and causes prolonged flooding (Auerbach et al., 2015), or lead to a net export of sediment to the open sea, resulting in land erosion and channel widening (Auerbach et al., 2015; Guchhait et al., 2016; Liu et al., 2018). Ecosystems in lower delta regions, such as mangrove forests, can be adversely affected by reduced sediment flux. When accretion rates within the forest are reduced, forests can drown if accretion rates fail to keep pace with sea level rise (Friess et al., 2019; Nienhuis et al., 2020). Sand mining, common in the Mekong River, also reduces sediment flux and exacerbates bank erosion (Hackney et al., 2020; Jia et al., 2007).

To diagnose the causes and consequences of human-induced changes to hydro-sediment dynamics (see Boxes 1 and 2), palaeolimnological research can detect shifts in water flow or flooding frequency by tracking ecological changes to phytoplankton communities or shifting nutrient regimes (Chen et al., 2018; Liu et al., 2012). For example, a study on the long-term trends in the hydrological regime of a shallow lake in the Magdalena River in Colombia based on phytoplankton and biogeochemical markers indicated that the existing lake shifted first from a flood-dominated riverine system to a connected wetland system, then shifted again to a lake system ~60 years ago (Lopera-Congote et al., 2021). This reduced hydrological connectivity, partly caused by land-use change around the lake, contributed to increased sediment run-off and eutrophication (Lopera-Congote et al., 2021). This study highlighted that the best management option was to increase connectivity with surrounding hydrology.

**Box 1.** Sediment dynamics and potential coring in Lakes.

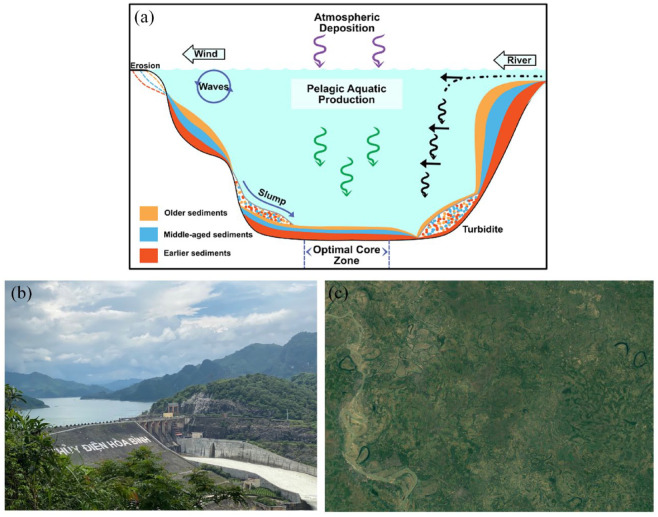 **Figure B1.** (a) Depiction of sediment deposition dynamics in an aquatic system. Image modified from Håkanson (1982). (b) Satellite image of oxbow lakes in the NW section of the Ganges-Brahmaputra-Meghna Delta system. Taken from Google Earth Pro. (c) Image of a reservoir on the Da River in the Red River Delta system, Vietnam. Photo Credit: Richard Walton. The forces behind sediment deposition in lakes are dynamic and dependent on a range of physical factors including hydrological in-and-out flow, bathymetry, atmospheric deposition, wind and wave action, biodeposition and bioturbation among others (Håkanson and Jansson, 1983; Manning, 2011; Schillereff et al., 2014). The dynamics of delta environments may make finding a suitable coring site and getting a reliable chronology somewhat more complicated than in other landscapes. For example, monsoonal rains in South and Southeast Asia may increase sedimental inflow from the surrounding landscape as well as forming seasonal wetlands of relatively shallow depth (Islam et al., 2021). Naturally shifting channels or anthropogenic water diversions may alter the amount of hydrological and sedimental input available to a lake during the dry season (Remondi et al., 2016; Zhang et al., 2018). Furthermore, the amount of biological productivity in a lake, especially in tropical regions, can increase organic deposition and the chances of bioturbation, or disturbance of the sediments from organismal activities (Salgado et al., 2020). This can complicate where coring for palaeolimnological research is able to take place.
Many deltas, fortunately, still have ample choice for suitable coring sites that should yield a robust chronology. Provided no scouring or dredging has occurred over the past several decades, oxbow lakes and reservoirs >2 m depth are ideal locations to core in tropical mega-deltas. Oxbow lakes, which are stranded river meanders, are common features of large river deltas. When not still largely connected to its mother river channel, the sediments that settle out in the deepest points of the lake may be able to capture many of the signals (Galbarczyk-Gąsiorowska et al., 2009; Gell and Reid, 2014; Kattel et al., 2016) that policymakers and resource managers would be interested in for a particular region as well as a wider landscape-scale view. Furthermore, reservoirs are also good locations where coring can be productive due to their connection with the wider river catchment and often deep areas that allow for sediment deposition to take place largely undisturbed (Chanudet et al., 2016; Fong et al., 2020; Wengrat et al., 2019). It should be noted, however, that reservoirs will only contain sediments from the point of construction and will therefore not capture any effects prior to major human activity in the region.

**Box 2.** The Yangtze River floodplain.

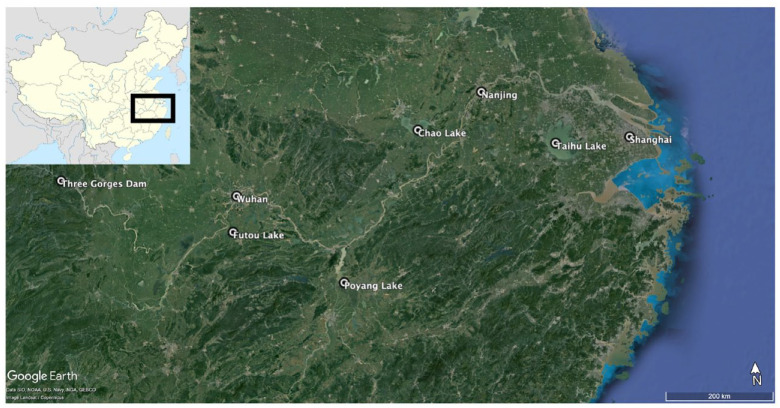 **Figure B2.** Map of Yangtze River middle and lower floodplains. Produced on Google Earth Pro. The Yangtze River floodplain encompasses portions of the middle and lower reaches of the river and includes many lakes and wetlands. Although this floodplain is not a tropical river delta, floodplains can act as good analogues for understanding lakes systems in tropical deltaic systems. Complex floodplains such as on the Yangtze can have similar dynamics to deltas, with lakes connected directly to river channels or through seasonal flooding that can rapidly flush the system (Dearing et al., 2012; Tockner et al., 1999) and subject it to accelerated taphonomic pressures (Bozelli et al., 2009; Heiler et al., 1995). Several palaeolimnological studies have shown that alterations in hydrological connectivity during the mid-20th and late-20th century has been the main driver for declines in water quality and increases in sedimentation rates in floodplain lakes (Xue and Yao, 2011; Xu et al., 2017; Yang et al., 2014; Zhang et al., 2018). In Lake Poyang, Zhang et al. (2018) found through diatom analysis that hydrology was the dominant driver of ecosystem health and functioning prior to changes in hydrological connectivity and supply between 1949 and 1995. After which due to significant land use change in the catchment, an upsurge of flow into the lake alongside a rise in nutrients, precipitating the eutrophication of the lake (Zhang et al., 2018). This was further compounded by the building of the upstream Three Gorges Dam which reduced hydrological connectivity (Zhang et al., 2018). Diatom analysis of four lakes along the Yangtze floodplain showed hydrodynamics were previously the driving force of their ecological status, but the lakes underwent eutrophication due to reduced hydrological connectivity after several dams were built upstream (Yang et al., 2014). More importantly for managers concerned with resources derived from floodplain lakes, smaller, shallow lakes and those with few macrophytes experienced the greatest acceleration in sedimentation rates arising from land-use change (Xue and Yao, 2011; Xu et al., 2017).

Palaeolimnology is particularly useful in understanding changes in hydrology and sediment flux. Shifts in sediment accumulation rates within a particular basin can be quantified by radiometric dating (Cohen, 2003; Foucher et al., 2021), and shifts in hydrological sources and connectivity of deltaic lakes can be understood through a range of techniques including grain-size analysis and geochemical or organic tracing of sediment sources (Collins et al., 2020). Both aid in understanding elemental point sources and how that has influenced ecological conditions (Bertrand et al., 2005; Liu et al., 2012; Salgado et al., 2020). While difficulties may exist in finding suitable coring sites (see Box 1), careful employment of palaeolimnology in tropical deltas should be able to better help managers understand how changing hydrology or sediment fluxes can impact their resources.

## Salinisation

Excessive saline intrusion into coastal water bodies is a growing problem in South and Southeast Asian delta communities (Clarke et al., 2018; Eslami et al., 2019; Szabo et al., 2016a). Increased instances of salinisation of soils and waterways are threatening livelihoods by reducing agricultural and aquacultural crop harvests, making coastal aquifers unusable and altering ecological functioning (Habiba et al., 2014; Hossain et al., 2016; Tran et al., 2022). Salinisation can be a gradual and irreversible process (caused by sea-level rise, salinisation of groundwater, or an excess of nutrients in floodplain agricultural fields) or can occur in temporary, periodic pulses (storm surges or seasonal droughts). The history of natural salinisation and the triggers for its occurrence and impacts in comparison with anthropogenically-driven salinisation need investigating as a key tool in managing this problem in these regions.

Fortunately, palaeolimnology and proxy indicators of salinity have been frequently employed (Table 1) and used to change policy on managing salinisation in coastal environments (Gell et al., 2007; Salgado et al., 2020). Reconstructing the history of change across multiple ecosystem components provides a much more robust understanding of salinity changes within the wider terrestrial-aquatic system (Gell et al., 2002; Reid et al., 2002; Smol and Stoermer, 2010). Palaeolimnological studies provide management insights into the vulnerability of lake and wetland systems to salinisation by examining the historical development of delta systems. For example, palaeolimnological studies have shown that Tonle Sap Lake originated as a brackish waterbody tied to the Mekong (Penny, 2006) and has tracked the response of mangrove coverage in Cambodia to shifts in salinity (Li et al., 2012) prior to the Mekong Delta progradation into modern-day Vietnam. Other studies have shown reductions in freshwater discharge are responsible for rising salinity in Brazilian and East Asian deltaic waterways (Castro et al., 2013; Cho et al., 2017; Zong et al., 2010b). Several studies have also linked shifts in salinity to specific, sudden events such as a storm surge event impacting deltaic lakes in the Arctic Circle (Deasley et al., 2012; Pisaric et al., 2011; Thienpont et al., 2015) leading to long-term ecological impacts, a scenario in which resource managers in tropical mega-delta systems may want to prepare for as such extreme events are argued to become more likely (Bevacqua et al., 2020; MacLeod et al., 2021; Rao et al., 2020).

**Table 1.** Ecological, geochemical and other environmental proxies used in palaeolimnology and how they can be used to understand the impacts of stressors on aquatic systems.

Sediment Proxy	Description of proxy	Variable (s) proxy used to reconstruct	Delta stressor variable can inform on	References
Diatoms	Cosmopolitan fossilised microscopic algae with silica cell walls (frustules). The different morphologies of diatoms make them easy to identify.	• Community compositional and abundance changes can be used to reconstruct changes in nutrient enrichment, salinity, pH and the thermal/light structure of lakes (e.g. through shifts in benthic to pelagic species) • Lake and hydrological evolution reconstructions using community compositional changes and stable isotopes from their silica cell walls (e.g. δ^18^O)	• Nutrient enrichment • Saline intrusion. • Natural to anthropogenic mediated hydrological alteration. • Industrial pollution • Natural to anthropogenic alterations to land-use • Natural to anthropogenic climate variability	Leng and Barker (2006), Duong et al. (2019), Briddon et al. (2020)
Algal pigments	The light absorbing compounds; chlorophylls and carotenoids of photosynthetic organisms	• Same as diatoms • Increased concentrations of cyanobacterial pigments could indicate the presence of cyanotoxins which are harmful to human and ecological health.	• Same as diatoms • Public health impacts of cyanotoxins through inferred abundance changes in cyanobacterial pigments.	Leavitt and Hodgson (2001), Waters et al. (2005)
Zooplankton remains: Chironomids and Cladocerans	The different morphologies of fossilised head capsules of non-biting midges (Chironomidae) and the chitinous remains of water fleas (Cladocera) which include the carapace, headshield, and appendages.	• Chironomid assemblage changes have been used to reconstruct air temperature (using chironomid-temperature transfer function), trophic status and water depth. • Cladocera are not always well-preserved but one of the only fossil representatives from the pelagic zone. Compositional and abundance changes are used to infer changes in pH, trophic status and water depth.	• Natural to anthropogenic climate change. • Natural to anthropogenic mediated hydrological alteration. • Nutrient enrichment	Brooks (2006); Wojewódka et al. (2020).
Ostracods	Fossilised calcium carbonate carapaces (shells) of the bi-valved crustacean which resemble water fleas. Ostracods are found in almost all aquatic habitats.	• Ratios of different geochemical elements and the stable isotope compositions of the ostracod shells can indicate changes to aquatic conditions • Presence/absence of species has been used to estimate past air temperature and salinity: The Mutual Ostracod Temperature Range (MOTR) and the Mutual Ostracod Salinity Range (MOSR).	• Saline intrusion. • Hydrological alterations • Temperature variability • Industrial pollution (heavy metals) • Oxygenation (changes to oxygen within a water body could be driven by hydrological change/lake ontogeny or development over time/anoxia events etc.)	Chivas et al. (1986), Gasse et al. (1987), Mischke et al. (2010)
Foraminifera (forams)	Single-celled protists whose shells are built of calcium carbonate (calcareous) or from tiny grains of sand stuck together (agglutinate)	Species compositional and shell geochemistry changes used to reconstruct changes in multiple environmental conditions such as salinity and dominant elemental composition.	• Saline intrusion • Natural to anthropogenic hydrological alterations • Land-use change • Nutrient enrichment	Scott and Medioli (1986), Benito et al. (2015)
Pollen	Microscopic fossilised male fertilising agents from plants, trees, grasses and weeds.	• Climate change using compositional changes • Environment changes through compositional and abundance changes (e.g. increase in pollen from agricultural crops indicate human land-use modification)	• Natural to anthropogenic climate variability • Natural to anthropogenic land-use change (e.g. conversion of mangrove forest to agriculture) • Natural to anthropogenic hydrological/water quality change	Bennett and Willis (2001), Hofmann (2002)
Plant macrofossils	Fossilised remains from vegetation that do not require microscopy to identify e.g. leaf, stem debris.	• Same as pollen	• Same as pollen	Birks (2001), Salgado et al. (2020)
Sediment grain size	The size of the grains within a sediment sample provides information on the composition, source, transportation and deposition of the sediment.	• Used to identify frequency and magnitude of flood events/the speed of water which determines the deposition of the sediment and the connectivity to the surrounding watershed.	• Flooding events • Natural to anthropogenic climate variability • Natural to anthropogenic alterations to watershed morphometry/hydrology	Tye and Coleman (1989), Liu et al. (2012), Chen et al. (2018)
Spheroidal carbonaceous particles (SCPs)	Distinct component of black carbon formed by the combustion of fossil fuels (coal and oil) at high temperatures (>1000°C).	Fossil fuel combustion	• Industrialisation • Urbanisation	Rose (2015), Engels et al. (2018)
Geochemical analysis: heavy metals and minerals.	Identifying the elemental composition of sediment using techniques including XRF (X-ray fluorescence)	• Concentrations and ratios of different elements can infer erosion and land-use change. • Increased concentrations of heavy metals can indicate industrial, sewerage and mining activity.	• Flooding events. • Natural to anthropogenic climate variability • Industrialisation (e.g. heavy metals) • Urbanisation • Mining activity	Last and Smol (2002), Vonk et al. (2015)
Stable isotopes from the sedimentary organic matter	Stable isotopes such as δ^15^N and δ^13^C can be used to determine the source of lake organic matter (e.g. terrestrial or allochthonous vs in-lake or autochthonous) and the trophic status of the lake. Measured using mass spectrometry.	• δ^15^N has been used to identify different N sources and processes of organic matter including sewerage and artificial fertiliser inputs, as well as N_2_-fixing cyanobacteria. • δ^13^C has been used to identify the productivity of lakes and inputs of terrestrial organic matter.	• Nutrient enrichment • Natural to human-mediated land-use change (e.g. conversion of mangrove forest to agriculture)	Meyers and Teranes (2001), Wengrat et al. (2018)
Sedimentation rates	Determined by measuring the radioactive nuclide signatures in the sediment such as ^210^Pb/^137^Cs etc.	• Changes to sedimentation rates can be used to reconstruct changes in sources of sediment and their transport. The deposition of sediments within a lake can also tell us about the thermal structure of the water column and its chemistry, its bathymetry and hydrological regime.	• Flooding events • Natural to human-mediated land-use change • Natural to anthropogenic climate variability • Natural to anthropogenic alterations to watershed morphometry/hydrology • Nutrient enrichment • Industrialisation • Urbanisation	Gell et al. (2009), Xu et al. (2017).

Palaeolimnology can help elucidate the impacts of recent and sudden events of salinisation in ecological systems, as well as putting these types of events into historical and climatic context. For example, Roberts et al. (2022) found that coastal lakes in eastern Norfolk, England, had been regularly inundated with seawater during past storm surge events. Recovery to pre-event conditions was dependent not only on the severity of the event, but also on how the intrusion event interacted with other drivers of ecological conditions such as sedimentation rates or ongoing modifications to the catchment, that is, agricultural drainage (Roberts et al., 2022). When combining modern sampling and monitoring techniques (see Pisaric et al., 2011; Thienpont et al., 2012) with local community knowledge (Kokelj et al., 2012), use of palaeolimnology to better track and understand the scale of salinity impacts in tropical deltas can offer a more holistic solution for resource managers and policymakers.

## Nutrient enrichment and other anthropogenic pollutants

Nutrient and pollutant levels play key roles in determining water quality within delta systems. Over the last ~70 years, anthropogenic impacts on biogeochemical cycling in aquatic systems (Rockström et al., 2009) have led to limits being set to avert widespread eutrophication of global marine and freshwater systems (Steffen et al., 2015). Eutrophication, from high nitrogen (N) and phosphorus (P) inputs, results in excessive primary production and can trigger shifts in aquatic community assemblages such as the onset of harmful algal blooms (Scheffer, 1989). Elevated levels of eutrophication may lead to reductions in aquatic biodiversity (Jeppesen et al., 2000; Sayer et al., 2010), which can surpass key ecological thresholds such as a clear water, macrophyte-dominated system becoming a turbid phytoplankton dominated system (Davidson and Jeppesen, 2013; Davidson et al., 2023; Jinglu et al., 2007). Freshwater and marine environments can respond differently to nutrient loading. Freshwater environments are typically P-limited (although see Lewis, 2000 for tropical exceptions) whilst marine environments are N-limited (Cloern, 2001; Pinto-coelho, 1998). Increasing reactive nitrogen pollution in tropical deltas (Lee et al., 2019) has led to an exacerbation in the total number of coastal eutrophication cases.

Nutrient sources arising from anthropogenic activities can be readily detected in tropical river systems, such as in the Red River Delta, Vietnam, where four nutrient point sources were detected using stable isotopes (Luu et al., 2020) and may be preserved in the sediment record as Fontana et al. (2014) revealed in their study that untreated sewage was the cause of eutrophication in a Brazilian reservoir. Moreover, stronger aquatic biogeochemical responses to eutrophication have been documented in tropical delta systems when compared with temperate regions (Corredor et al., 1999; Downing et al., 1999). As well as (now scarce) unmodified natural lake systems, reservoirs have proven useful in reconstructing nutrient enrichment over more recent timescales due to their importance as reliable public water resources and known period of creation (Lewis, 2000). Studies in three tropical reservoirs in Brazil have shown that prolonged eutrophication over several decades has altered and simplified the phytoplankton community, with cascading impacts on other species (Costa-Böddeker et al., 2012; Wengrat et al., 2018) and with increased occurrences of toxic cyanobacterial blooms (Fontana et al., 2014).

A common impact of eutrophication is the shift in planktonic communities to frequent and intense cyanobacteria blooms (Labaut et al., 2020; O’Neil et al., 2012; Taranu et al., 2015). These blooms can lead to losses in aquatic biodiversity, reduced attenuation of light in the water column for photosynthetic organisms, and bottom water hypoxia triggering fish kills and lakebed internal loading. As a result, lakes can become locked into turbid states (Merel et al., 2013; Visser et al., 2016). Blooms also have the capacity to produce harmful algal toxins (e.g. hepatoxins and neurotoxins) causing Harmful Algal Blooms (HABs) in high concentrations. Algal pigment biomarkers or photosynthetic sulphur bacteria (for identifying lake anoxia) in sediments (Table 1) can identify the relative proportion of algal groups over time (e.g. eukaryotic algae (diatoms, chlorophytes, cryptophytes), cyanobacteria and photosynthetic bacteria) and their response to anthropogenic climate change and pollution. The consequences of increased phytoplankton productivity in response to eutrophication can favour cyanobacteria and mixotrophic algal groups due to resource competition and light availability (Gangi et al., 2020).

Reconstructing historical eutrophication and its associated effects on the timing and rate of ecosystem deterioration is valuable for informing tangible management plans. For instance, palaeolimnological methods can be used to determine the nutrient sequestration rates by coring reservoirs upstream of deltas and verify estimates of nutrient starvation caused by damming (Quynh et al., 2005). Alternatively, Wengrat et al. (2019) were able to separate natural trophic variability within five Brazilian reservoirs and showed that eutrophication of each reservoir was a gradual process with varying levels of ecological impact. In this instance, the authors were able to advise managers that not all trophic changes in the reservoirs were a response to anthropogenic impacts, and the first several years of a reservoir’s existence are too unstable to be used as reference conditions, with the most effective management of the reservoirs would come from a catchment-scale approach (Wengrat et al., 2019).

As well as nutrient enrichment, the rise of the Anthropocene has also seen the increasing transmission of organic and inorganic pollutants (ranging from metals, pesticides, fungicides, persistent organic pollutants (POPs), personal care products (PCPs), polycyclic aromatic hydrocarbons (PAHs) and other chemical compounds into aquatic ecosystems. Pollution events and their subsequent effects can often be clearly detected in the sediment record. For example, Costa-Böddeker et al. (2018) found elevated levels of Pb, Cr and Ni amongst other metal pollutants accumulating in the last two decades in the Thi Vai Estuary, Vietnam due to recent industrialisation and changes in land use. In Central America, Salgado et al. (2020) also found that the construction of the Panama Canal led to increasing pollutant delivery in Gatun Lake typically associated with increased mining and fossil fuel combustion such as zinc (Zn), copper (Cu) and lead (Pb). Coupling the timing of pollutants entering the system with collapse ecological communities through palaeolimnology can provide the necessary evidence needed to introduce regulation into the system (Sayer et al., 2006).

Mangrove forests are also indicator environments of pollutants in tropical deltas because they sequester material transported from both land and sea. Pollutant trapping is a recognised ecosystem service potentially acting as an important policy driver for the restoration and protection of these valuable forests. By reducing pollutant concentrations in the water column, wider ecosystem health is improved (Vo et al., 2012). Palaeolimnological methods employed on more recent sediments may help assess changes in the severity of pollution over time and determine whether measures to reduce pollution release are working. For example, treaties on halting atmospheric testing of thermonuclear weapons have resulted in a sharp decrease of anthropogenic radionuclide pollution since the 1970s (Walling et al., 1995), although continued testing in some areas of the tropical Pacific may provide distinct pollution signatures (Chaboche et al., 2022) during the 1970s phase out. Use of palaeolimnology in mangrove ecosystems has been used to track heavy metal and hydrocarbon contaminants from boats, cars and effluent (Costa et al., 2015), as well as coastal eutrophication (reconstructed by diatom species assemblage and C:N ratio change (Logan and Taffs, 2009; Macreadie et al., 2012)), and microplastic pollution (Deng et al., 2021). These methods can then be used to identify the main sources for the pollution (such as urbanised areas, sewage outflows, tourism activities and ocean circulation), and recommend on steps needed to control pollution levels and provide remediation (Wu et al., 2014). The extent of transboundary impacts relative to those arising from local pollution sources, however, remains unknown for many tropical river systems, making palaeolimnology a key tool to answer such questions.

## Multiple stressors

The key issues discussed above are often considered in isolation by policymakers and resource managers. Aquatic ecology and fluvial dynamics are, however, complex systems driven by multiple stressors, resulting in a wide array of challenges to tropical delta resilience (Renaud et al., 2013; Szabo et al., 2016b; Woodroffe et al., 2006). As we show here, palaeolimnology can help resource managers understand the impact of multiple stressors at landscape-scales, in a more holistic way. Crucially, it can interpret sediment records from both terrestrial and aquatic stressors within the delta catchment (Gell et al., 2009; Hunt and Rushworth, 2005; Wengrat et al., 2019) to disentangle multiple impacts (Anderson et al., 2006; Smol, 2010) and assist resource managers and policy makers in implementing the best solutions (see Box 3), particularly when multiple basins are compared across regions to find primary stressors (Mills et al., 2017).

**Box 3.** Palaeolimnology and management.

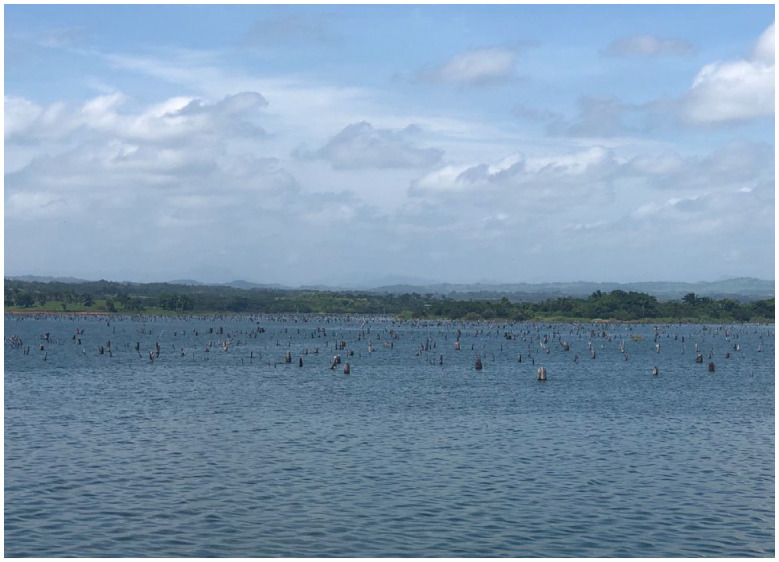 **Figure B3.** Gatun Lake in Panama. Photo credit: Jorge Salgado. Palaeolimnology has been a key informer of management plans in recent decades. For example, it has played an important role in the development of an ecosystem-based risk management plan to understand the environmental controls on salinity in two South American coastal lagoons important to the regional economy (Velez et al., 2018). In palaeolimnological research used to reconstruct the systems over the last 10,000 years, it was shown that hydrological connectivity was the determining factor for salinity levels in the lagoons, with freshwater discharge and changes to sea-level over time being the major determinants of lagoon salinity (Inda et al., 2016; Van der Hammen and Noldus, 1984; Velez et al., 2014). As such, managers were encouraged to place hydrological connectivity at the top of the list of the priorities for maintaining the SES (Velez et al., 2018). Another study using a multi-proxy palaeolimnological approach, revealed that a century after the Chagres River was impounded to build the Panama Canal, salt intrusions gradually increased in the coastal Gatun Lake, one of the largest freshwater sources for Panamanians (Salgado et al., 2020). These changes in salinity were evident from observations in increases in salinity-tolerant and marine diatom species along with increases in calcium concentrations. In Brazil, reservoirs such as the Guarapiranga and other neighbouring impoundments (Fontana et al., 2014; Gangi et al., 2020; Wengrat et al., 2019) are among the most prominent examples of palaeolimnological research influencing resource management. Here, palaeoecological studies showed reductions in diatom species diversity over recent decades was due to a response to cultural eutrophication, derived from untreated industrial and urban effluent sources as identified by increasing stable isotope compositions of nitrogen (d^15^N) in the sediment record (Fontana et al., 2014; Wengrat et al., 2018). The research highlighted in these studies have provided well-grounded recommendations for the effective management of tropical reservoir systems, including the provision of well-defined baseline conditions and records of past productivity, whilst highlighting the significance of constraining wider basin scale changes and analytical tools to assess ecological change (Wengrat et al., 2019). Such examples clearly demonstrate how palaeolimnology studies can lead to management plans that are better suited for environmental recovery and adaptation while allowing these regions continue to find ways increase sustainability in natural resource extraction.

Many different anthropogenic activities are influencing deltaic changes in hydrology, sediment supply, soil and water salinity and nutrient/heavy metal pollution, such that managing a single stressor is unlikely to resolve the matter (Jackson et al., 2016; Vinebrooke et al., 2004). Palaeolimnological studies have helped understand how multiple stressors can be synergistic or antagonistic over time to create the physical and ecological issues we observe in the present. For example, Salgado et al. (2019) studied the effects of historical changes of native aquatic plants to lake surface area, eutrophication and the invasion of water hyacinth (*Eichhornia crassippes)* in Lake Fúquene, Colombia. By using multiple cores and a multi-proxy approach (plant macrofossils, pollen and trace elements) the study found that the increase in invasive hyacinth was triggered by lake management practices (diversion of hydrological inflow) acting synergistically with internal and continued external nutrient loading leading to eutrophication, benefitting the spread of water hyacinth over native species.

Elsewhere, a study in Lake Furnas on São Miguel Island, Azores, Portugal identified that eutrophication, warming water column temperatures and anthropogenically introduced fish species in the 19th century, could not explain present-day anoxia in the system. Only the co-occurrence of the three stressors, in addition to the introduction of larger piscivorous fish in the late-20th century, could explain phytoplankton community changes that depleted the already-reduced levels of dissolved oxygen creating anoxic conditions (Buchaca et al., 2011). Both above examples pre-dated monitoring of those systems meaning that palaeolimnology could fill in the data gaps and help to better explain multiple stressors on deltaic systems (Salgado et al., 2020; Smol, 2010; Wengrat et al., 2019).

## Conclusion

The stresses on tropical river deltas and their communities, both naturally occurring and anthropogenically induced, constitute a major threat to tropical river delta SESs. Many processes such as changes to hydrology and sediment supply, ingress of saline waters and excessive inputs of nutrients and pollutants occur at timescales that are either too extended or precede the onset of monitoring. Environmental data availability is limited in many delta regions, which endangers the ability for delta ecosystems and human communities to proceed in a resilient and sustainable manner. Palaeolimnological methods can address the problems of data scarcity in delta regions, enabling managers to set reference conditions for remediation and to better understand the long-term dynamics driving the scale of impact from individual and multiple stressors. Commissioning the use of palaeolimnology in the updating of policy or management plans can provide the critical long-term data on sensitivity and change that is key to making such action well-informed and effective. We therefore strongly encourage governments, resource managers and scientific experts alike to more fully use palaeolimnological tools and advocate for their inclusion at the outset of any remediation efforts in these regions.
